# Development of the Sexual Minority Adolescent Stress Inventory

**DOI:** 10.3389/fpsyg.2018.00319

**Published:** 2018-03-15

**Authors:** Sheree M. Schrager, Jeremy T. Goldbach, Mary Rose Mamey

**Affiliations:** ^1^Office of Research and Sponsored Programs, California State University, Northridge, Northridge, CA, United States; ^2^Division of Hospital Medicine, Children's Hospital Los Angeles, Los Angeles, Los Angeles, CA, United States; ^3^Suzanne Dworak-Peck School of Social Work, University of Southern California, Los Angeles, CA, United States

**Keywords:** adolescents, LGBT, minority stress, measure development, item response theory

## Abstract

Although construct measurement is critical to explanatory research and intervention efforts, rigorous measure development remains a notable challenge. For example, though the primary theoretical model for understanding health disparities among sexual minority (e.g., lesbian, gay, bisexual) adolescents is minority stress theory, nearly all published studies of this population rely on minority stress measures with poor psychometric properties and development procedures. In response, we developed the Sexual Minority Adolescent Stress Inventory (SMASI) with *N* = 346 diverse adolescents ages 14–17, using a comprehensive approach to de novo measure development designed to produce a measure with desirable psychometric properties. After exploratory factor analysis on 102 candidate items informed by a modified Delphi process, we applied item response theory techniques to the remaining 72 items. Discrimination and difficulty parameters and item characteristic curves were estimated overall, within each of 12 initially derived factors, and across demographic subgroups. Two items were removed for excessive discrimination and three were removed following reliability analysis. The measure demonstrated configural and scalar invariance for gender and age; a three-item factor was excluded for demonstrating substantial differences by sexual identity and race/ethnicity. The final 64-item measure comprised 11 subscales and demonstrated excellent overall (α = 0.98), subscale (α range 0.75–0.96), and test–retest (scale *r* > 0.99; subscale *r* range 0.89–0.99) reliabilities. Subscales represented a mix of proximal and distal stressors, including domains of internalized homonegativity, identity management, intersectionality, and negative expectancies (proximal) and social marginalization, family rejection, homonegative climate, homonegative communication, negative disclosure experiences, religion, and work domains (distal). Thus, the SMASI development process illustrates a method to incorporate information from multiple sources, including item response theory models, to guide item selection in building a psychometrically sound measure. We posit that similar methods can be used to improve construct measurement across all areas of psychological research, particularly in areas where a strong theoretical framework exists but existing measures are limited.

## Introduction

Sexual minority adolescents (SMA; lesbian, gay, bisexual, pansexual, etc.) experience well-documented behavioral health disparities compared to their heterosexual peers. In addition to reporting lower academic achievement (D'Augelli et al., 2002; Kosciw et al., 2012); key outcomes for which SMA are at notably elevated risk include internalizing and externalizing disorders (Fergusson et al., 1999); internalizing psychopathology, such as depression, anxiety, and self-harm (Anhalt and Morris, 1998; Haas et al., 2011; Hendricks and Testa, 2012); eating disorders and obesity (Austin et al., 2013); and substance use (Marshal et al., 2009). SMA are also more than twice as likely to have attempted suicide than their heterosexual counterparts (Marshal et al., 2013), with as many as 42% of SMA having considered suicide at some point in their life (D'Augelli et al., 2001). When disparities such as these emerge in adolescence, they can negatively influence a lifelong trajectory of health and well-being (Steinberg and Morris, 2001).

The primary framework for understanding the mental health disparities experienced by SMA is minority stress theory (MST; Meyer, 2003). MST suggests that discrimination, violence, and victimization due to a pervasive homophobic culture are the primary sources of stress and most probable driving mechanisms of mental health problems among sexual minorities (Savin-Williams, 2001; Russell, 2003; Kelleher, 2009). The theory posits that general circumstances in the environment (including socioeconomic circumstances and living conditions) and minority status (including sexual orientation, race, ethnicity, and gender) are interconnected. As Meyer (2003) explains, individuals in a minority group—in this case, sexual minorities—are affected by two sets of minority-related stressors. These include both distal stressors in the environment, such as prejudicial events, discrimination, and violence, and proximal stressors internal to the individual, including expectations of rejection, concealment, and internalized homophobia. Research has suggested that these factors are chronic and unique in their contribution to mental health (Rosario et al., 2002). It is important to note that these factors are interrelated and bidirectional. For example, having a negative disclosure experience (distal) may increase expectations of rejection (proximal). At the same time, concealing one's identity due to rejection expectations (proximal) may reduce the likelihood of victimization; research has suggested that disclosing a sexual minority identity, or “being out,” to more individuals is correlated with higher rates of violence and victimization (Chesir-Teran and Hughes, 2009; Kosciw et al., 2015).

MST represents the first theoretical perspective to present a comprehensive explanatory framework for social-behavioral problems in this population. Nonetheless, numerous cross-sectional studies have suggested that disparities in behavioral health outcomes among SMA may be attributable to minority stress experiences, including disclosure of sexual identity to family and peers (Remafedi et al., 1998; Almeida et al., 2009; D'Augelli et al., 2010; Haas et al., 2011), fear of becoming homeless upon disclosure (Rice et al., 2013), bullying and victimization in school (Russell et al., 2011; Toomey et al., 2011; Kosciw et al., 2013), and other experiences of violence (Friedman et al., 2011).

Unfortunately, meta-analyses (Marshal et al., 2008; Goldbach et al., 2014) have shown that studies of minority stress during adolescence have been fraught with poor psychometric measurement and frequently rely on “home-grown” measures with poor psychometric reliability. In particular, few studies have used empirically validated measures, and most measures had been developed using small investigator-led samples or adapted from measures with adults in other minority populations. These methodological choices have led directly to inconsistencies in the empirical literature; for example, studies of SMA that have relied on measures of general distress tended to find large correlations with substance use (*r* = 0.60), whereas those using adapted or investigator-led measures of minority stress found a much weaker relationship (*r* = 0.24; Goldbach et al., 2014). Thus, previously available measures of minority stress among SMA may not accurately capture the stress–outcome relationship: General measures may capture more breadth, but do not allow us to differentiate between common developmental stressors and those associated with minority stress, yet measures specific to the constructs described by MST are lacking for adolescents.

These concerns were further highlighted in a recent review of psychometric measurements assessing discrimination against sexual minorities (Morrison et al., 2016), which found that among 162 articles, nearly all reported suboptimal psychometric properties. For example, only 13 (9.4%) of articles tested scale dimensionality, only five (3.1%) clearly articulated how they assessed content validity, only three (1.9%) assessed criterion-related validity, and only four (2.5%) explicitly tested construct validity. Only one measure achieved a “perfect score” of five points: The Daily Heterosexist Experiences Questionnaire (Balsam et al., 2013). Although a well-designed measure of minority stress with nine subscales, this measure was not intended for use with adolescents and describes numerous experiences that are not relevant to SMA.

This gap in measurement poses substantial challenges to explanatory research and intervention development efforts (Sandler et al., 1997), hampering our ability to model or address the theoretical determinants of mental health disparities among SMA. In response, the present study sought to develop a new measure of minority stress, the Sexual Minority Adolescent Stress Inventory (SMASI), through empirically rigorous methods. The current paper describes in detail the development of this measure using a multiphase design and several complementary analytical approaches to data reduction.

### Preliminary studies

#### Qualitative study

The development of an initial pool of candidate items for the SMASI emerged from a multiphase qualitative study of SMA (Goldbach and Gibbs, 2015, 2017). Following a formative assessment with 25 key informants at three local organizations that serve a large number of SMA, a sample of 48 SMA aged 13–19 from diverse sexual orientation, gender, and racial and ethnic groups participated in a 90- to 120-min semi-structured life history calendar interview. Based on the extant literature on both domains of minority stress (e.g., Meyer, 2003; Hatzenbuehler, 2011; Goldbach et al., 2014) and existing instruments used in previous research, the research team identified nine key domains for SMA (life landmarks; sexual minority milestones; sexual identity expressions; home life; peer group; school; spirituality; race and ethnicity; and community connection). Thematic analysis (Boyatzis, 1998) of the transcribed interviews yielded 125 distinct stress experiences in those nine domains that formed the basis of the first version of the SMASI.

#### Modified delphi process

Although the initial qualitative study provided an excellent foundation for an adolescent-focused minority stress measure, important decisions about the measure concerning item framing, response options, and time frame to measure, among other topics, remained. To incorporate expert opinion on these topics in a rigorous, structured way, a modified expert panel study employing the RAND/UCLA Appropriateness Method, or Delphi process, was conducted (Fitch et al., 2001). This phase of the study is described in detail elsewhere (Schrager and Goldbach, 2017). In brief, an advisory panel of six experts in diverse SMA, MST, and stress measure development rated all candidate items for feasibility and face validity; participated in three half-day video conferences to discuss their ratings, suggest new items and content areas, and make recommendations about item structure; and rated all remaining and newly developed items for validity and feasibility again. After two rounds of ratings, the initial list of 125 candidate items informed by the qualitative interview data yielded a final testable measure comprising 104 items and representing 12 unique minority stress domains. Two items describing physical and sexual assault experiences were excluded to conform to institutional review board requirements regarding mandated reporting of abuse to children, given the safeguards to protect participant confidentiality in our survey study. The final candidate item set as tested (Table 1) included 102 items representing 12 initial content domains: four proximal (sexual identity, gender identity, disclosure, internalized homonegativity) and eight distal (family, school, peers, neighborhood, religion, racial/ethnic community, work, and connection to the broader LGBT community).

**Table 1:** Candidate items for the sexual minority adolescent stress inventory (as tested).

**Item**	**Item stem**
1	I feel like I should act more “straight.”
2	Sometimes I date people of the opposite sex in order to make others think I'm straight.
3	I feel like an outcast because I am LGBTQ.
4^*^	I am questioning how to label my sexual orientation.
5^*^	I am having trouble accepting that I am LGBTQ.
6^*^	I feel pressured to label myself as gay or lesbian.
7^*^	I am concerned that if I am LGBTQ, I will have a worse life than if I were straight.
8	I have been told that I am LGBTQ because something bad must have happened to me when I was younger.
9	Being LGBTQ makes me feel like “less of a [man/woman].”
10	I have been made fun of for acting like a [girl/boy].
11	I want to come out to my family but don't know how.
12^*^	A family member told other family members that I am LGBTQ without my permission.
13^*^	A family member told me not to tell other family members that I am LGBTQ.
14^*^	I have to lie to my family about being LGBTQ.
15^*^	I think I will lose friends if I come out as LGBTQ.
16	I have acted “straight” in order to keep friends.
17^*^	I expect people to reject me when they find out that I am LGBTQ.
18	Someone has rejected me after finding out that I am LGBTQ.
19^*^	If I come out, it will cause problems within my family.
20^*^	A family member asked me if I was gay or lesbian before I wanted to talk about it.
21^*^	I was forced to come out to someone because I got “caught.”
22^*^	I was “outed” by someone other than my family without my permission.
23	I have denied being LGBTQ after being asked.
24^*^	There are times when I do not want to be LGBTQ.
25^*^	If I could, I would become straight.
26^*^	I hate being LGBTQ.
27^*^	I think it is wrong for me to be LGBTQ.
28^*^	I hope that being LGBTQ is just a phase for me.
29^*^	I think negatively about other LGBTQ people who act “too gay.”
30^*^	I am uncomfortable with being LGBTQ.
31^*^	I have heard a family member make negative comments about LGBTQ people.
32^*^	My mother (or female caregiver) does not accept me as LGBTQ.
33^*^	Someone who lives with me has told me they disapprove of me being LGBTQ.
34^*^	I feel as though I am a disappointment to my family because I am LGBTQ.
35^*^	My family has told me that being LGBTQ is just a phase.
36^*^	My parents are uncomfortable with LGBTQ people.
37^*^	My father (or male caregiver) does not accept me as LGBTQ.
38	One or more of my siblings does not accept me as LGBTQ.
39^*^	My family does not want to talk to me about being LGBTQ.
40	I have been called bad names or slurs by a family member because I am LGBTQ.
41^*^	My parents are sad that I am LGBTQ.
42	My family treats me worse than my sibling(s) because I am LGBTQ.
43^*^	My family tries to make me straight.
44^*^	I have felt unsafe or threatened in school because I am LGBTQ.
45	I have had to leave or change schools because I am LGBTQ.
46	I have felt isolated or alone at school because I am LGBTQ.
47	I have lost friendships since coming out as LGBTQ at school.
48	I have been physically assaulted by students at school because I am LGBTQ.
49^*^	Other youth refuse to do school activities with me because I am LGBTQ.
50^*^	I have seen other LGBTQ youth treated badly at my school.
51^*^	It's hard to be an LGBTQ person at my school.
52^*^	Other students make fun of me for being LGBTQ.
53	My school does not protect LGBTQ students.
54	A teacher or staff at my school is unsupportive of me because I am LGBTQ.
55	A teacher or staff at my school is unwilling to stand up for me.
56	In school, LGBTQ topics are not covered at all in classes.
57^*^	I have seen other LGBTQ youth treated badly at work.
58^*^	I have felt unsafe or threatened at work because I am LGBTQ.
59^*^	I have had to leave or change jobs because I am LGBTQ.
60^*^	I have felt isolated or alone at work because I am LGBTQ.
61^*^	I have lost friendships since coming out as LGBTQ at work.
62^*^	It's hard to be LGBTQ at my workplace.
63	Coworkers harass me for being LGBTQ.
64^*^	I have been physically assaulted by people at work because I am LGBTQ.
65^*^	My workplace does not protect LGBTQ employees.
66^*^	People at work talk about me being LGBTQ behind my back.
67^*^	My boss is unsupportive of me because I am LGBTQ.
68^*^	I have seen other LGBTQ youth treated badly in the neighborhood where I live.
69^*^	I have felt unsafe or threatened in the neighborhood where I live because I am LGBTQ.
70^*^	I have had to move or change where I live because I am LGBTQ.
71^*^	I have felt isolated or alone in the neighborhood where I live because I am LGBTQ.
72^*^	Other people in the neighborhood where I live make fun of me for being LGBTQ.
73^*^	I have been physically assaulted in the neighborhood where I live because I am LGBTQ.
74	I feel uncomfortable in bathrooms/locker rooms because I am LGBTQ.
75^*^	My friends make jokes about LGBTQ people.
76	My friend's parents are not accepting of me being LGBTQ.
77	My friends try to convince me I am not really LGBTQ.
78^*^	Other youth refuse to hang out with me because I am LGBTQ.
79^*^	Other people who are in my racial/ethnic community judge me for being LGBTQ.
80	I have seen other LGBTQ people being treated badly by people in my racial/ethnic community.
81^*^	I have heard negative comments from others in my racial/ethnic community about being LGBTQ.
82	Someone in my racial/ethnic community has told me that being LGBTQ is not okay.
83^*^	I feel as though I don't fit in my racial/ethnic community because I am LGBTQ.
84^*^	As an LGBTQ person in my racial/ethnic community, I feel like I am a minority within a minority.
85	I believe that it is harder to be an LGBTQ person of color than to be an LGBTQ white person.
86	In general, I don't like the LGBTQ community.
87	I don't have a connection to the LGBTQ community.
88	I cannot relate to people in the LGBTQ community.
89	The LGBTQ community is hard to access in my area.
90	I wish I had an LGBTQ role model.
91^*^	I hear other LGBTQ people use words like “fag” or “dyke.”
92	There are parts of the LGBTQ community who do not accept me.
93	I feel as though the LGBTQ community favors white gay men.
94	There is a lot of “in-fighting” between different groups within the LGBTQ community.
95	I feel like my religious community or church would not want me there because I am a LGBTQ.
96^*^	My family is part of a religion that has homophobic beliefs.
97^*^	I have heard negative messages about being LGBTQ from religious people.
98^*^	I would not be accepted as an LGBTQ person in my family's religion.
99	I feel like if you are LGBTQ you cannot be religious.
100^*^	I believe it is wrong for me to be LGBTQ because of my religion.
101^*^	A religious leader has encouraged me to reconsider my sexual orientation.
102^*^	A religious leader tried to change my sexual orientation.

*LGBTQ, lesbian, gay, bisexual, transgender, or queer*.

* *Retained in the final SMASI measure*.

## Materials and methods

### Participants and recruitment

Recruitment of participants was conducted between February 2015 and July 2016. Eligibility criteria included residence in the United States; age between 14 and 17 years; self-identification as cisgender male or female; self-identification as lesbian, gay, bisexual, or pansexual, or willingness to endorse one of these terms describing sexual identity for youth who preferred another label; and willingness to provide electronic assent to participate in research. Twenty initial participants, who also served as seeds in a respondent-driven sampling framework (Heckathorn, 2011), were recruited from two agencies serving LGBTQ adolescents in Los Angeles County and an additional event serving LGBTQ youth. Subsequently, recruitment transitioned to Internet-based methods, using targeted advertisements on Facebook and investigator-initiated posts on Reddit in a discussion sub-forum (“r/LGBTeens”) frequented by the population of interest. Simultaneously, targeted advertising of the survey expanded from urban centers in California (Los Angeles, San Francisco) to nationwide. Regardless of recruitment method, all study participants were invited to generate a unique referral code to refer other sexual minority youth to the study, and peer recruitment chains constituted the remainder of the study sample. Participants received $25 for participation in the survey study and an additional $10 for every eligible participant they referred.

**Figure 1:**
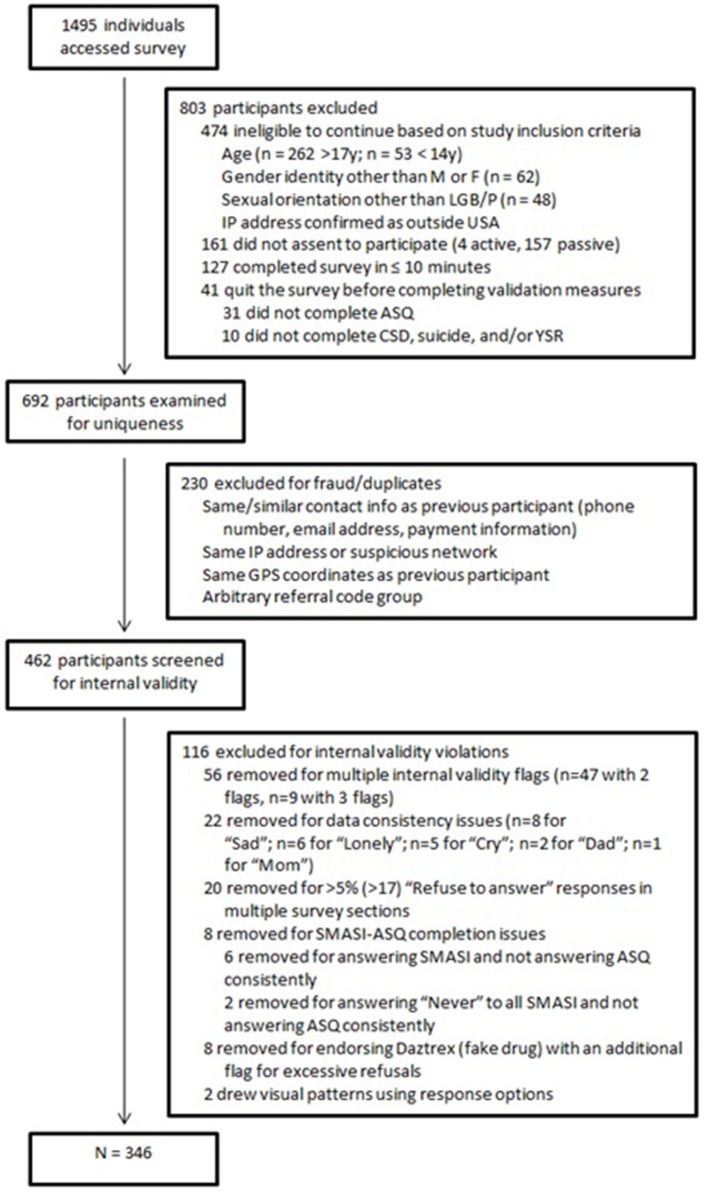
CONSORT enrollment diagram.

Figure 1 presents the flow diagram of participant recruitment and exclusion. Of the 1,495 individuals who attempted to access the survey, 635 did not meet study eligibility criteria (including not indicating assent to participate) and were prevented from accessing the study survey. Another 41 accessed the survey but did not validly complete one or more key measures, including the minority stress instrument under study, and thus could not be included in the analytic sample. Finally, a detailed analysis of potential fraud and internal validity was conducted, and additional participants were identified as “malicious responders” (Robinson-Cimpian, 2014) on one or more internal indices of data quality. Specifically, following the methods of Aust et al. (2013), we removed participants who were confirmed duplicates of previous participants via repeated email or IP address (*n* = 230); who demonstrated “exceedingly short completion times” (Aust et al., 2013, p. 528)—in the present study, 10 min or less (*n* = 127); or due to serious inconsistencies in their responses to different measures (*n* = 116). A final sample of 346 participants was retained for subsequent analysis. Demographic characteristics of these participants are presented in Table 2.

**Table 2:** Participant demographics.

	***n* (%)**
**GENDER**	
Male	151 (43.6)
Female	195 (56.4)
**AGE**	
14	35 (10.1)
15	84 (24.3)
16	114 (32.9)
17	113 (32.7)
**RACE AND ETHNICITY**	
Non-hispanic white	144 (41.6)
Black or African American	40 (11.6)
Latino or hispanic	84 (24.3)
Asian	28 (8.1)
American Indian or Alaska Native	16 (4.6)
Native Hawaiian or other Pacific Islander	5 (1.4)
Other	3 (0.9)
Multiracial	26 (7.5)
**SEXUAL ORIENTATION**	
Gay	147 (42.5)
Lesbian	107 (30.9)
Bisexual or pansexual	92 (26.6)
**ENROLLED IN SCHOOL**	
Yes	335 (96.8)
No	11 (3.2)
**EMPLOYMENT STATUS**	
Full-time	8 (2.3)
Part-time	48 (13.9)
Not employed but previously worked	45 (13.0)
Not employed and never worked	244 (70.5)
**PRIMARY LANGUAGE AT HOME**	
English	294 (85.0)
Spanish	40 (11.6)
Other	12 (3.5)
**PRIMARY LANGUAGE WITH FRIENDS**	
English	341 (98.6)
Spanish	4 (1.2)
Other	1 (0.3)
**REFERRAL SOURCE**	
In-person recruitment event	26 (7.5)
Facebook ad	73 (21.1)
Reddit ad	22 (6.4)
Respondent-driven sampling chain	220 (63.6)
Unclear or invalid entry	5 (1.4)

### Study procedures

Potential in-person participants were recruited by agency staff and referred to a member of the research team in a private room on-site who obtained verbal assent to participate. Those recruited through social media (Facebook, Reddit) were asked to participate by clicking on the provided link or advertisement. These participants were initially routed through a gatekeeping web page to prevent duplicated IP addresses from accessing the main survey repeatedly. Participants with unique IP addresses were automatically redirected to the main survey in the Qualtrics platform (Qualtrics, 2017), which used a responsive design format that optimized the display based on the type of device accessing the survey and permitted the use of piped language and branching logic based on prior survey responses. Participants recruited in person completed the Qualtrics survey on tablets provided by the study team, whereas participants recruited online completed the survey at their leisure on a computer or mobile device (e.g., tablet or smartphone).

Upon accessing the main Qualtrics survey, participants were first asked to enter a referral code, used for paying referrers in the respondent-driven sampling chain, or for tracking recruitment source if the participant had responded directly to an online advertisement. Participants then completed the eligibility screening items (including age, gender, sexual identity, and ZIP code). Eligible participants were shown the study assent form and were asked to indicate their assent to participate via a single binary item before continuing.

At the end of the survey, participants had the opportunity to download a list of resources for support tailored to SMA. They were then redirected to a separate payment page to collect payment information without the possibility of including potentially identifiable contact information, including email address, in the main study dataset. After providing their email address for payment (a $25 online gift card), participants had the opportunity to generate a referral code that they could use to recruit other youth into the study.

A subset of 24 participants who had agreed to participate in a follow-up survey and provided contact information was approached by email for participation in the test–retest study 2 weeks after they completed the main survey. Of these individuals, 22 completed the follow-up survey, which included only the eligibility screener (for text piping purposes) and the 102-item SMASI. Based on the same criteria as the main study, seven of these participants were excluded, resulting in a 15-person test–retest sample with complete data. All study procedures were approved by the institutional review board at the authors' affiliated university.

### Measures

Upon accessing the survey, participants first completed the screening items (age, gender identity, sexual identity, and ZIP code) to assess and verify eligibility; after qualifying and providing assent to participate, they completed several additional demographic items including school status, work status, and language preference.

The primary measure of interest was presented immediately after the demographics section and consisted of the 102 candidate items comprising the draft SMASI. The initial item set presented statements nominally reflecting minority stress experiences (e.g., “My parents are sad that I am LGBTQ”) and asked participants to indicate when this experience happened to them by checking all time frames that applied. Possible response options were *never, within the past 30 days, more than 30 days but less than 3 months ago, more than 3 months but less than 6 months ago, more than 6 months but less than 1 year ago*, and *more than 1 year ago*. If “never” was chosen, participants were prevented from selecting any other time points. Participants also had the option to decline to answer the question. If at least one response other than “never or “refuse to answer was chosen—i.e., the participant affirmatively indicated experiencing the stressor at least once—a follow-up question asked, “The last time this happened to you, how stressful was it?” Responses were on a 5-point Likert-type scale ranging from 1 = *not at all stressful* to 5 = *very stressful*. Scoring of the SMASI is discussed in considerably more detail in the Recoding SMASI Items section, below. Two items were programmed based on the gender endorsed by the participant: “I have been made fun of for acting like a [girl/boy]” and “Being LGBTQ makes me feel like less of a [man/woman],” wherein male participants were shown “girl” and “man,” respectively, and female participants were shown “boy” and “woman.” A set of 11 items describing stress experiences at work were only answered by participants who reported that they were currently employed full-time or part-time or unemployed but had worked previously; participants who reported having never worked were not shown these items.

Because a secondary aim of the study was to test the validity of the emerging measure (Goldbach et al., 2017), researchers were also interested in collecting information related to other sources of stress and mental and behavioral health. For this reason, the survey also included the Adolescent Stress Questionnaire (Byrne et al., 2007), the abbreviated (56-item) Revised Youth Self Report (Ivanova et al., 2007), the four-item version of the Center for Epidemiologic Studies Depression Scale (Melchior et al., 1993), an additional three items probing suicidality and self-harm, and 18 items assessing lifetime and past-30 day substance use from the Youth Risk Behavior Survey (CDC, 2010).

### General approach to data analysis

A series of analytic approaches was used to thoroughly investigate the utility, strength, and importance of the 102 candidate items of the SMASI measure. First, analyses of variance (ANOVAs) were conducted to investigate the relationship between recency and stress and determine which response time frames to use in the remaining analyses. Next, exploratory factor analysis (EFA) was conducted to understand the underlying structure of the measure. Item response theory (IRT) analyses were then conducted for the overall scale and subscales to assess each item's difficulty and discrimination parameters. Invariance across four demographic groupings (i.e., gender, sexual identity, race and ethnicity, and age) was tested to determine whether any items functioned differently for different subgroups of participants. Reliability testing, using Cronbach's alpha for cross-sectional data and correlations for the 2 week longitudinal data, was performed for the overall scale and by subscale. Finally, we examined correlations among the SMASI subscales to ascertain the degree of uniqueness or overlap among the retained factors. At each step of analysis, items that did not make both statistical and theoretical sense were eliminated from the measure, and the subsequent analysis used only the remaining items. For all analyses involving significance testing, *p*-values were adjusted based on the Benjamini and Hochberg (1995) false discovery rate controlling procedure to limit the likelihood of basing decisions about item retention on potentially spurious findings.

## Results

### Recoding SMASI items

For the purposes of examining recency effects on stress, the SMASI time frame items were converted into indicators of recency by capturing the most recent occurrence of an experience (among one or more affirmative responses) for each item. For each item, the most recent time frame selected was recoded into an ordinal variable representing the (0 = *never*, 1 = *past 30 days*, 2 = *30 days to 3 months*, 3 = *3 to 6 months*, 4 = *6 months to 1 year*, 5 = *more than 1 year ago*). For example, if “more than 30 days but less than 3 months ago” and “more than 1 year ago” were both selected for a given item, the value of the corresponding recency item would be 2, reflecting only “more than 30 days but less than 3 months.” This variable was created to provide context for the follow-up items capturing how stressful an experience was the last time (i.e., the most recent time) that it occurred.

One-way between-subjects ANOVAs were used to examine the relationship between recency and level of stress reported by the participants. Among the 102 recency-coded items, only 18 (17.6%) demonstrated a significant association with the reported stressfulness of that item after adjusting for multiple comparisons. These items were not conceptually linked to one another. Furthermore, *post-hoc* tests revealed that when significant differences in stressfulness emerged by recency of the event, they tended to occur when participants who evaluated a stressor that most recently happened a year ago or longer reported the experience as being more stressful than participants who experienced it at other time points, particularly those who had experienced that stressor during the past 30 days.

Therefore, we chose to move forward with two binary codings of the stress experience items: the first set of items reflected whether a person had experienced the specific item (situation) in his or her lifetime, and the second set of items described whether a person had experienced the situation in the past 30 days. Lifetime binary items were created by assigning a value of 1 (*yes*) to those who endorsed at least one of the time frames for a given item and 0 (*no*) to those who selected “never” for that item. Past-30 day binary items were created using a similar process; those who endorsed the “within the past 30 days” timeframe for a given item were assigned a value of 1 (*yes*) and those who did not select this option for the given item were assigned 0 (*no*). Given the previous ANOVA results, variables reflecting 30 day and lifetime stressors were assumed to sufficiently represent minority stress experiences in this population.

Of note, an explicit assumption was made that over the course of a lifetime—even an adolescent lifetime—certain experiences would cohere. For example, if a respondent's family had been unsupportive of their sexual identity in one way, such as having made jokes about LGBTQ people, they would be very likely to have been unsupportive in other ways, such as saying their LGBTQ identification is just a phase. However, it is not equally realistic to expect, if a family member made one specific unsupportive comment within the past 30 days, that they would also have made those other unsupportive comments in that same time frame. Thus, although it is reasonable to require stability and good psychometric performance from the lifetime items, we believed the 30 day items would not be as informative in generating a stable, useable measure that would be replicable over time and in other samples. For this reason, we conducted the scale and subscale development analyses, including EFA, IRT, and reliability analysis, on the lifetime data only. The results of these analyses are presented below.

### Exploratory factor analysis

Factor analysis of the lifetime binary items was conducted in SPSS using the principal components analysis extraction method per convention with dichotomous variables (Meulman et al., 2004). The initial model specified direct oblimin (oblique) rotation to allow for factors to be correlated. Given recent cautions against conducting parallel analysis with dichotomous variables (e.g., Tran and Formann, 2009), we relied on the typical approach to EFA, in which factors with an eigenvalue greater than 1.00 were retained in the factor structure. Missing values due to participant refusal to answer were replaced using idiographic mean substitution across the entire measure, because listwise and pairwise deletion would result in eliminating too many cases or failure to converge. An a priori requirement for item retention was a moderate loading (0.40) onto one factor; items that did not load moderately onto one factor and items that loaded at or above 0.40 onto multiple factors were eliminated.

The EFA of the 102 lifetime binary SMASI items resulted in an initial 14-factor measure (Supplementary data sheet 1). The component correlation matrix was subsequently examined to determine whether an orthogonal or oblique rotation would be more appropriate for the data. Correlations of 0.32 between factors correspond to approximately 10% of variance overlap; factors with this value or greater should be correlated using an oblique rotation (Tabachnick and Fidell, 2007). Because only five of the factor correlations resulted in values greater than 0.32—and no correlations were greater than 0.40—the EFA of the binary lifetime variables was re-run using the principal component extraction method with equamax rotation, reflecting the assumption of orthogonality. Equamax rotation maximizes factor loadings onto one factor while minimizing factor loadings onto the other factors (Meulman et al., 2004). This resulted in a 14-factor measure with interpretable factor groupings and loadings (Supplementary data sheet 2). Nineteen items were eliminated at this step because they did not have a sufficiently large loading (≥0.40) onto any factor. Six items were eliminated because they loaded onto more than one factor. One item was eliminated because it was the only item that loaded onto a factor. Finally, one factor consisting of four items was eliminated at this stage because it consisted only of items reflecting beliefs rather than stress experiences *per se* and did not add theoretical meaning to the measure. The remaining 12 subscales, comprised of 72 items, were subjected to an additional round of EFAs to verify unidimensionality within each proposed subscale. As only one factor per subscale was found, all 72 items were moved forward to IRT analyses. Local independence could not be explicitly assessed, but was assumed from the absence of patterned responses (i.e., local linear trends) on visual inspection of the data. Finally, EFA was conducted in Mplus Version 7.11 (Muthén and Muthén, 2013) to verify the stability of this factor structure, using the WLSMV estimator to appropriately model the binary lifetime stress indicators.

### Item response theory

The goal of IRT is to focus on specific items rather than the total score and is an important step in scale creation to understand the underlying properties of each item. Each of the following analyses used a two-parameter logistic model (2PL) to understand both difficulty and discrimination values of each item. In the context of the current study, an item's difficulty can be viewed as the amount of the underlying experience—in this case, minority stress overall or within a given domain—required for a person to have a 50% probability of endorsing the item. Small difficulty values suggest that the item is easily endorsed, whereas larger difficulty values suggest more experience of the underlying construct is needed to endorse the item. Items can also differ in terms of their discrimination. An item's discrimination level is essentially a measure of how well the item differentiates respondents based on the difficulty parameter. High discrimination values suggest that the item is better able to differentiate respondents who will later report higher or lower minority stress levels, whereas smaller discrimination values suggest less efficiency in this regard. The item discrimination parameter in IRT is similar to the item factor loading in factor analysis. An individual's response to each of the binary items in the SMASI was examined based on the individual's trait level, item difficulty, and item discrimination.

IRT analyses were estimated for the measure overall, within factors, and across groups (gender, sexual identity, race and ethnicity, and age), also using Mplus Version 7.11 (Muthén and Muthén, 2013). Item functioning was investigated by examining difficulty and discrimination parameter values, item content, and item characteristic curves. The following analyses helped guide decisions about item retention or elimination, although final decisions also incorporated theoretical justification.

#### Overall

**Figure 2:**
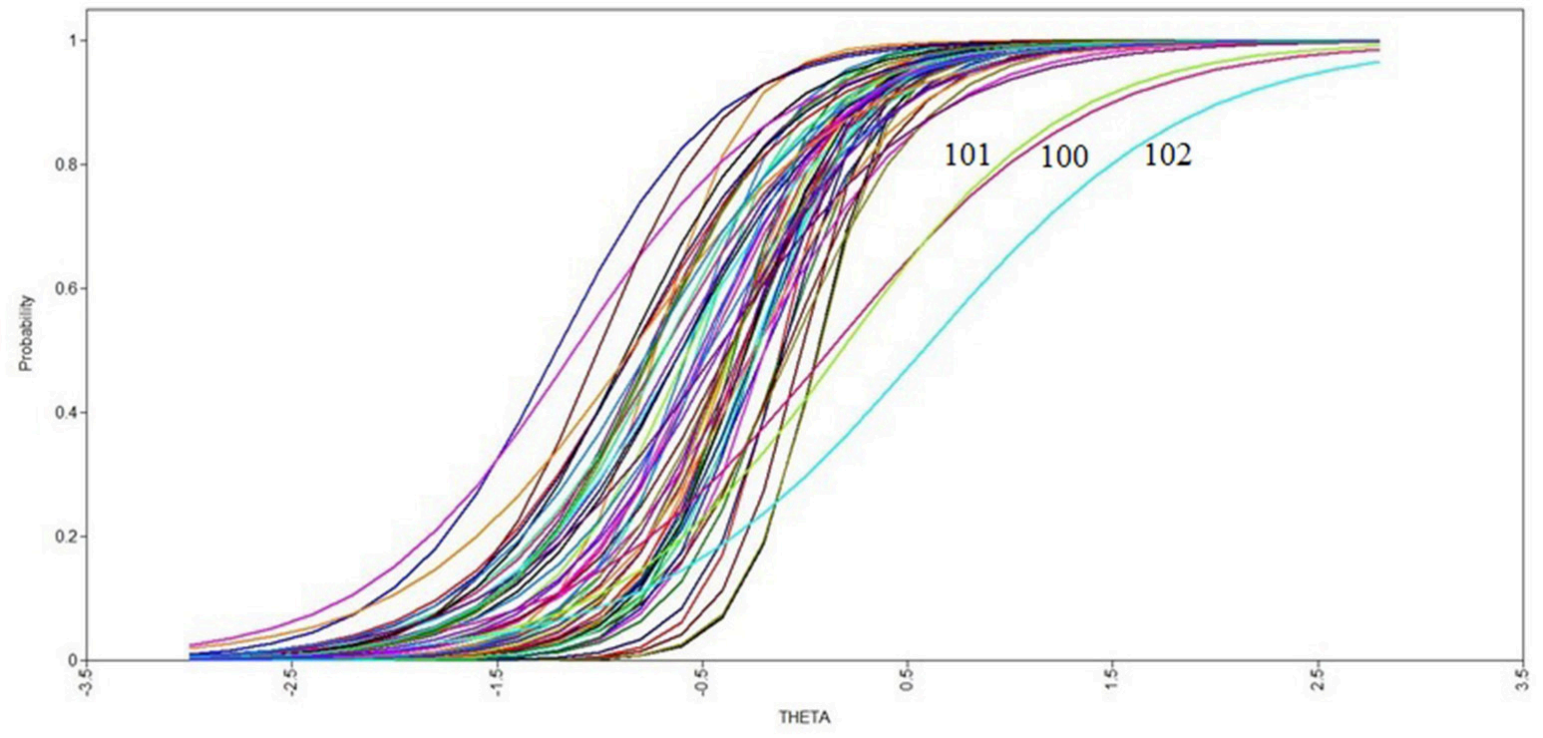
Item characteristic curves from overall item response theory analysis. (Numbers of curves with extreme values represent item numbers from Table 1).

The SMASI at this stage consisted of 72 candidate items. Because only participants with a history of employment had access to items that formed a unique factor describing stressors at work, these 11 items were excluded from the IRT analyses of the overall scale, allowing responses from all participants to the remaining 61 items to be used in the overall IRT analyses. The item characteristic curves from this analysis are depicted in Figure 2. The difficulty values for the SMASI items ranged from −1.210 to 0.572 standard deviations surrounding the mean on the minority stress latent variable. The usual range of the difficulty parameter is between −2 and 2; thus, our results suggest that it takes a relatively small amount of the underlying experience (i.e., adolescent minority stress) to endorse the majority of these items, as reflected in the x-axis placement of each of the 61 slopes in Figure 2. The discrimination values of the SMASI items ranged from 1.512 to 5.668 above the mean. The usual range of the discrimination parameter is between 0.5 and 2.5, suggesting high discrimination ability among a majority of the minority stress items. This is reflected in the slopes in Figure 2, wherein items with higher discrimination have a steeper slope. Although the discrimination values were moderately high, no single item was eliminated from the measure after this analysis.

#### By factor

IRT analyses were also performed within each of the 12 factors to allow for further investigation of the items underlying a specified latent trait.

##### Social marginalization

This factor consisted of eight items. The difficulty parameters ranged from −0.072 to 0.196 and the discrimination parameters ranged from 4.651 to 12.120. One item with a discrimination value of 12.120 (“Other people in the neighborhood where I live make fun of me for being LGBTQ”) was not removed, because the nature of the question warrants a high discrimination value due to its specificity. No items were eliminated in the social marginalization factor.

##### Family rejection

This factor consisted of 11 items and had acceptable difficulty (−0.717 to −0.127) and discrimination (2.749–5.030) parameter values. All items remained in the family rejection factor.

##### Internalized homonegativity

The seven items demonstrated an acceptable difficulty range of −0.499 to 0.016 and an acceptable discrimination range of 2.635–7.358. No items were removed from this factor.

##### Identity management

All four items had acceptable difficulty (−1.082 to −0.392), but the range of discrimination was 2.072–443.203. Two items (“I am questioning how to label my sexual orientation” and “There is a lot of ‘in-fighting’ between different groups within the LGBTQ community”) had values of 301.884 and 443.203, respectively. The latter item was removed for reflecting more of a belief than a true stress experience, and the analysis was re-run. The removal of this item resulted in acceptable difficulty (−0.536 to −0.379) and discrimination (2.462–5.068) values for all remaining items.

##### Homonegative climate

This factor consisted of four items, each of which had acceptable difficulty (−0.496 to −0.091) and discrimination (2.810–5.214) values. No items were eliminated.

##### Intersectionality

This factor consisted of three items, all of which had acceptable difficulty (−2.336 to −0.787) and discrimination (3.915–6.513) values. No items were eliminated.

##### Negative disclosure experiences

The eight items of this factor had acceptable difficulty (−0.505 to −0.111) and discrimination (2.853–3.922) parameter values. No items were eliminated.

##### Religion

The five items of this factor remained in the measure, with acceptable difficulty and discrimination values of −0.354 to 0.453 and 2.378–4.483, respectively.

##### Homonegative communication

All six items of this factor had acceptable difficulty (−1.238 to −0.752) and discrimination (1.828–3.699) levels. No items were eliminated.

##### Negative expectancies

The four items comprising this factor had good difficulty (−0.791 to −0.314) and discrimination (2.545–4.608) values. No items were eliminated.

##### Work

This factor consisted of 11 items, with difficulty parameters ranging from 0.418 to 1.078 and discrimination parameters ranging from 2.254 to 16.167. The item with a discrimination value of 16.167 (“Coworkers harass me because I am LGBTQ”) was removed and the analysis was re-run with the remaining 10 items, resulting in a difficulty range of 0.417–1.054 and a discrimination range of 2.254–7.364. The 10-item work factor was retained.

##### Concealment

The final factor consisted of three items, each of which had good difficulty and discrimination levels of −0.781 to −0.285 and 1.780–3.805, respectively. No items were eliminated at this stage.

The IRT analyses for these 12 factors resulted in the elimination of two items, with 60 items remaining in the main SMASI measure and 70 items when including the work factor.

### Invariance testing

Invariance was tested across gender, sexual identity, race and ethnicity, and age to determine whether any item functioned differently as a result of membership in other demographic subgroups. The change in CFI between configural (non-restrictive) and scalar (restrictive) models were observed to determine whether the more restrictive model (i.e., constrained factor structure, loadings, and thresholds) resulted in a decrement in fit as compared to the baseline model (i.e., unconstrained factor structure). ΔCFI >0.01 would result in further examination of thresholds and loadings (parameters) across groups to understand whether substantive differences in responses existed across groups for a given item. Chi-square difference tests were also conducted and reported, though due to their sensitivity to smaller sample sizes, ΔCFI was used as the primary criterion to test measurement invariance. ΔRMSEA was also examined as a secondary criterion.

#### Gender

Gender (male vs. female) demonstrated both configural and scalar invariance for the majority of the 12 factors. Internalized homonegativity [$\Delta \chi_{\left(13\right)}^{2}$ = 27.85, *p* < 0.05], religion [$\Delta \chi_{\left(9\right)}^{2}$ = 18.88, *p* < 0.05], and the concealment factor [$\Delta \chi_{\left(5\right)}^{2}$ = 12.94, *p* < 0.05] showed a decrement in fit as loadings were constrained equal. Examination of the parameters both with and without constraints as well as the overall global fit of each constrained model suggested adequate fit between the models and the observed data (CFI = 0.987–0.996, TLI = 0.987–0.996, RMSEA = 0.079–0.097) for factors with a decrement in fit. No items were eliminated in this step.

#### Sexual orientation

Invariance was tested for sexual orientation by grouping individuals who identified as gay, lesbian, or bisexual. The identity management, negative expectancies, and work factors demonstrated invariance across groups as models moved from baseline to constrained models. However, chi-square difference tests showed decrements in fit for the remaining factors [social marginalization: $\Delta \chi_{\left(31\right)}^{2}$ = 70.40, *p* < 0.001; family rejection: $\Delta \chi_{\left(43\right)}^{2}$ = 155.83, *p* < 0.001; internalized homonegativity: $\Delta \chi_{\left(27\right)}^{2}$ = 85.58, *p* < 0.001; homonegative climate: $\Delta \chi_{\left(15\right)}^{2}$ = 34.27, *p* < 0.01; intersectionality: $\Delta \chi_{\left(11\right)}^{2}$ = 41.66, *p* < 0.001; negative disclosure experiences: $\Delta \chi_{\left(23\right)}^{2}$ = 123.19, *p* < 0.001; religion: $\Delta \chi_{\left(19\right)}^{2}$ = 60.78, *p* < 0.001; homonegative communication: $\Delta \chi_{\left(23\right)}^{2}$ = 123.26, *p* < 0.001; concealment factor: $\Delta \chi_{\left(11\right)}^{2}$ = 45.13, *p* < 0.001]. Both global and localized fit were examined for each of these factors. Each factor demonstrated adequate global fit (CFI = 0.960–0.998, TLI = 0.963–0.998, RMSEA = 0.079–0.159), and only the concealment factor had localized ill fit. Specifically, the concealment factor structure did not hold when constraining loadings and thresholds for the lesbian and bisexual groups, suggesting that the factor was conceptually different for those who identified as gay when compared to those who identified as lesbian or bisexual.

#### Race and ethnicity

Race and ethnicity variables were categorized for this analysis as White, Black, Hispanic, and Other. We found similar findings for race and ethnicity as we did with sexual orientation. Only the work factor was invariant across race [$\Delta \chi_{\left(59\right)}^{2}$ = 74.59, *p* > 0.05]; the other factors did not demonstrate invariance across race. However, examination of parameters revealed adequate localized and global fit for all factors (CFI = 0.939–0.996, TLI = 0.954–0.998, RMSEA = 0.088–0.199) except the concealment factor (CFI = 0.903, TLI = 0.935, RMSEA = 0.211). Specifically, the item “I feel like I should act more ‘straight’” for the Black, Hispanic, and Other groups showed poor fit within its factor structure.

#### Age

The same invariance tests for each factor were conducted across age, categorize as 14, 15, 16, and 17 years. Chi-square difference tests were significant for all factors except work [$\Delta \chi_{\left(59\right)}^{2}$ = 64.77, *p* > 0.05]. However, the remaining factors demonstrated adequate global fit (CFI = 0.982–0.998, TLI = 0.985–0.998, RMSEA = 0.048–0.146) and no localized ill fit.

The concealment factor was eliminated after this step. The problems associated with this factor were likely due to subgroup differences in response patterns, suggesting that individuals may experience or perceive the three stressors in this factor differently depending on their racial, ethnic, and sexual identities.

### Reliability analyses

#### Internal consistency

Cronbach's alpha values, including subscale recalculations with each item deleted, were subsequently computed for the overall SMASI measure (excluding the work factor) and for each of the 11 retained subscales. However, the appropriateness of this statistic to real-world data has been called into question (e.g., McNeish, 2017). Several of the assumptions underlying the alpha statistic, including tau equivalence, are rarely met, and in such cases alpha may underrepresent internal consistency. For this reason, we also computed the composite reliability (CR) index (Raykov, 1997, 2004), an alternative approach to estimating scale reliability that relaxes the assumption of tau equivalence.

Although no items were removed for statistically weakening the measure, three items were removed at this stage for conceptual or theoretical reasons to improve the coherence of the measure and its subscales. “I have been called bad names or slurs by a family member because I am LGBTQ” was removed from the negative disclosure experiences factor to promote a cleaner distinction between this factor and family rejection (on which it did not load as highly). “I feel like an outcast because I am LGBTQ” was removed from the negative expectancies factor, despite a slight decrement in alpha as a result, to preserve the utility of that factor as a measure of expected or anticipated rather than experienced rejection. Finally, “I have denied being LGBTQ after being asked” was eliminated from the homonegative communications factor, which subsequently retained only items reflecting verbal statements communicated by other people and directed at or overheard by the respondent.

Reliabilities for the final measure are presented in Table 3, along with descriptive statistics for the lifetime and 30 day scale scores. Overall reliability of the main 54-item measure, excluding items from the optional work factor, was excellent, with Cronbach's alpha = 0.98 and composite reliability >0.99. The 11 subscales, including work, had good to excellent reliabilities as well, ranging from α = 0.75/CR = 0.91 (negative expectancies) to α = 0.96/CR = 0.99 (social marginalization). Overall and subscale alpha reliabilities were also examined by subgroup; composite reliabilities, which require confirmatory factor analysis loadings that were not calculated within subgroups, were not. Excellent reliability for the overall measure was retained (all α = 0.97–0.99 by age, gender, sexual identity, and racial and ethnic subgroups). Some subscales showed minor variations in reliability by demographic subgroup; in particular, the negative expectancies factor performed somewhat worse among 17-year-olds (α = 0.66) than any other age group (age 14: α = 0.78; age 15: α = 0.83; age 16: α = 0.76). Homonegative communications also performed noticeably differently by sexual identity (gay and bisexual: α = 0.68; lesbian: α = 0.91) and age (age 14: α = 0.68; age 15: α = 0.84; age 16: α = 0.79; age 17: α = 0.84). No other subscales demonstrated Cronbach's alphas below 0.70 for any demographic subgroup.

**Table 3:** Reliabilities and descriptive statistics for the final SMASI.

	**Cronbach's α**	**Composite Reliability**	**Lifetime *M* (*SD*)**	**30 days *M* (*SD*)**
Overall scale (range 0–54)	0.983	0.996	31.53 (17.75)	10.39 (8.73)
Social marginalization	0.963	0.991	46.42 (44.24)	12.35 (18.73)
Family rejection	0.947	0.980	62.88 (37.58)	20.98 (24.92)
Internalized homonegativity	0.923	0.976	55.24 (40.34)	17.49 (20.89)
Identity management	0.793	0.922	66.23 (39.81)	21.53 (27.06)
Homonegative climate	0.864	0.949	58.12 (41.23)	17.85 (25.02)
Intersectionality	0.880	0.972	58.33 (44.11)	20.66 (29.61)
Negative disclosure experiences	0.857	0.943	58.90 (39.16)	13.70 (20.39)
Religion	0.856	0.961	49.97 (38.54)	17.41 (22.39)
Negative expectancies	0.753	0.907	68.74 (37.85)	23.84 (30.52)
Homonegative communication	0.814	0.925	78.59 (30.67)	34.29 (31.52)
Work	0.937	0.984	24.52 (34.12)	10.55 (18.27)

#### Test–retest reliability

The first set of comparisons examined consistency over time for each person in the small longitudinal subset (*N* = 15) by item for the 54 items comprising the main SMASI instrument. An insufficient number of participants in the test–retest sample reported a history of employment (*n* = 5), so test–retest results are not reported for the work factor.

Participants who either endorsed the same time frame for both the initial survey (T1) and the 2 week follow-up (T2) or endorsed a time frame at T1 that immediately preceded a timeframe endorsed at T2 were considered to be “consistent” in their responses for that item. Consistencies were first measured within items, capturing how many people had responded with consistency to that item. Items had a consistency range between eight and 15 (53–100%), suggesting a majority of participants were able to respond consistently to all retained items across a 2 week window. Additionally, within-person consistency scores were created for each participant in the test–retest sample and ranged from 26 (48.1%; moderate consistency) to 54 (100%; full consistency), wherein higher percentages demonstrate higher personal consistency over time. Again, results suggest that adolescents are able to respond consistently to the SMASI over time.

Test–retest reliability was also assessed with correlations between stress subscales and overall scores at T1 and T2. For the purposes of this analysis, subscale scores were calculated for the lifetime and 30 day binary item set by taking the mean of the items retained for each factor and multiplying by 100, giving the percentage of endorsed (i.e., experienced) items on that scale. Overall lifetime and 30 day scores were calculated as a sum of all lifetime and 30 day binary items, respectively, comprising the main 54-item measure. The overall score correlation between lifetime T1 and T2 was *r* = 0.995, *p* < 0.001, and between past-30 day T1 and T2 was *r* = 0.750, *p* < 0.01. Lifetime subscale correlations (Table 4, diagonal) ranged from 0.89 to 0.99 (all *p* < 0.01) and 30 day subscale correlations (Table 5, diagonal) ranged from 0.51 to 0.89.

**Table 4:** Correlations among lifetime SMASI subscales.

	**1**	**2**	**3**	**4**	**5**	**6**	**7**	**8**	**9**	**10**	**11**
1. Social marginalization	0.987										
2. Family rejection	0.730	0.979									
3. Internalized homonegativity	0.753	0.714	0.980								
4. Identity management	0.602	0.606	0.710	0.900							
5. Homonegative climate	0.822	0.687	0.732	0.560	0.964						
6. Intersectionality	0.759	0.726	0.665	0.498	0.705	0.967					
7. Negative disclosure experiences	0.781	0.743	0.650	0.540	0.689	0.690	0.949				
8. Religion	0.722	0.739	0.617	0.459	0.646	0.684	0.699	0.892			
9. Negative expectancies	0.586	0.582	0.626	0.580	0.591	0.516	0.536	0.491	0.984		
10. Homonegative communication	0.519	0.685	0.580	0.525	0.571	0.580	0.586	0.574	0.408	0.978	
11. Work	0.800	0.606	0.538	0.379	0.637	0.569	0.538	0.649	0.453	0.309	–

*Test–retest correlations (N = 15) presented on the diagonal*.

*All correlations are significant at the p < 0.001 level*.

**Table 5:** Correlations among 30 day SMASI subscales.

	**1**	**2**	**3**	**4**	**5**	**6**	**7**	**8**	**9**	**10**	**11**
1. Social marginalization	0.667^*^										
2. Family rejection	0.414^***^	0.810^***^									
3. Internalized homonegativity	0.445^***^	0.406^***^	0.718^**^								
4. Identity management	0.266^***^	0.325^***^	0.426^***^	0.664^*^							
5. Homonegative climate	0.593^***^	0.383^***^	0.458^***^	0.269^***^	0.894^***^						
6. Intersectionality	0.354^***^	0.344^***^	0.358^***^	0.132^*^	0.364^***^	0.511					
7. Negative disclosure experiences	0.495^***^	0.448^***^	0.477^***^	0.292^***^	0.432^***^	0.285^***^	0.572^*^				
8. Religion	0.339^***^	0.514^***^	0.306^***^	0.148^**^	0.348^***^	0.404^***^	0.391^***^	0.800^***^			
9. Negative expectancies	0.292^***^	0.451^***^	0.440^***^	0.352^***^	0.435^***^	0.358^***^	0.310^***^	0.296^***^	0.384		
10. Homonegative communication	0.205^***^	0.488^***^	0.312^***^	0.303^***^	0.304^***^	0.387^***^	0.151^**^	0.433^***^	0.355^***^	0.474	
11. Work	0.656^***^	0.375^***^	0.487^***^	0.413^***^	0.557^***^	0.377^***^	0.605^***^	0.366^***^	0.446^***^	0.192	–
											

Test–retest correlations (N = 15) presented on the diagonal

* p < 0.05;

** p < 0.01;

*** *p < 0.001*.

#### Subscale correlations

Correlations among the subscales are also presented in Table 4 (lifetime) and Table 5 (30 day). All lifetime subscales were significantly correlated at *p* < 0.001 with each of the other subscales. The past 30 day subscales were also significantly correlated with each other at *p* < 0.05, except for the correlation between homonegative communication and work (*r* = 0.19, *p* > 0.05).

## Discussion

### General methodological approach

Using the development of the SMASI as a proof of concept, the current study illustrated the utility of a comprehensive, multifaceted approach to de novo psychological construct measure development when no validated measures exist. Although our study, like many, began with qualitative pilot studies and investigator input to generate and refine items, we further developed our measure by incorporating expert opinion through a formal modified Delphi process, which expanded the content areas addressed by our measure from nine to 12. Subsequently, we tested the new item set in a diverse sample from the target population and applied multiple analytic approaches, including comparison of different response coding schemes, EFA, IRT, and calculation of traditional psychometric measures, to fully capture and explicate the underlying structure and performance of each tested item. Ultimately, this process resulted in the creation of a 54-item, 10-factor measure of minority stress, plus an optional 10-item subscale specific to youth in the workforce, that is reliable and face valid for use with diverse U.S. adolescents aged 14–17 and can be expected to function well despite regional and national demographic differences in that population.

Of particular interest in this study is the use of an IRT approach to assessing item performance overall and within demographic subgroups. Historically, IRT has been used in the context of educational test construction, with the assumption that some underlying latent trait (e.g., knowledge) accounts for differences in scores among test takers (Ghiselli, 1981), and the language of IRT models—e.g., difficulty and discrimination parameters—reflects this traditional use. More recently, IRT has been used to develop physiological or psychological assessments, moving out of the educational realm but still within the universe of scores that assume an underlying, intrapersonal trait, such as a health condition or disorder, for the assessment to confirm or deny (e.g., Hays et al., 2006; DeWitt et al., 2011). The current study, to our knowledge, represents the first attempt to apply IRT principles to the development of a measure of a purely theoretical social psychological construct without objective, previously defined criteria for its presence, absence, or level. Results suggest IRT is a flexible and highly beneficial tool for this type of use; in our study, the IRT procedures resulted in the exclusion of 12 items that would otherwise have been retained after traditional EFA, including an entire three-item factor that repeatedly demonstrated ill fit at the invariance testing phase. Incorporation of IRT procedures and results thus assisted us in developing a measure that is more likely both to assess minority stress experiences consistently across members of different demographic subgroups and to demonstrate good psychometric performance overall and within these subgroups in the future.

Traditional approaches to scale development were also used in the current study, with at times surprising results. ANOVAs to examine the perceived stressfulness of the proposed stressor items informed the decision to move forward with a binary operationalization of the measure, because perceived stressfulness largely did not differ by the time period in which it was last experienced. EFA revealed that contrary to expectations based on the qualitative pilot study, stress experiences were not neatly organized by location or source of the stressor (e.g., family, school, neighborhood, etc.) Instead, the emerging factors were better understood as manifestations of different psychological processes theorized to play a role in the experience of minority stress, including social marginalization from multiple sources, internalized homonegativity (formerly described as internalized homophobia; cf. Mayfield, 2001, p. 54), negative expectancies about future events, and so on. Of note, the largest number of items excluded during any stage of the scale development process, 30 items, occurred during the factor analysis step. Determining the function of those items that did not cohere to any well-defined factor in understanding the consequences of minority stress for adolescent mental and behavioral health is beyond the scope of this analysis, but remains an important question to investigate in future work.

### Online sampling and data selection

The final analytic sample (*N* = 346) represents only a quarter of the recorded attempts to complete the online survey. Although the majority of those failed attempts involved individuals outside the target population who were ineligible to participate, a non-trivial amount of the recorded data were excluded prior to analysis due to strong evidence calling the internal validity of the data into question. This is a typical finding in studies of adolescents (Fan et al., 2006; Robinson-Cimpian, 2014), and our final sample retention is in line with typical internet-driven sampling efforts. Considering that estimates of non-serious responding in online self-reported surveys range from 5% up to potentially 50% of responses (Aust et al., 2013), and given that this was a measure development study, the authors felt that erring on the side of excluding untrustworthy but potentially legitimate data was preferable to including all possible data and potentially creating an instrument with poor performance or that is unlikely to replicate well in legitimate samples. For this reason, we employed the methods described by Robinson-Cimpian (2014) and Aust et al. (2013) to ensure—prior to any measure development or outcome analysis—that we had strong evidence that our sample legitimately reflected our target population of U.S. sexual minority adolescents. Still, we acknowledge that this may have inadvertently biased our sample toward participants better able to complete the 30- to 45-min survey in a consistent manner, and the generalizability of our sample to other samples of SMA may be correspondingly limited as a result.

The small sample size is especially evident with our longitudinal test-retest sample. Given the large correlations predicted for test-retest reliability, *a priori* power analysis indicated that a very small test-retest sample of 22 participants would adequately power those analyses, even though this is virtually too small to calculate retest reliabilities (for which minimum sample sizes between 30 and 100 are typically recommended). Furthermore, although 22 participants were initially recruited to complete the 2 week follow-up survey, only 15 were included after the rigorous data screening processes described in the previous paragraph. We do note that the 15 test-retest participants were representative of the diversity in the overall sample (e.g., 53% age 14–15 and 47% age 16–17; 33% male and 67% female; 20% gay, 53% lesbian, and 27% bisexual/pansexual; 33% white, 47% Hispanic, and 20% other race). Lack of statistical power for correlations does not appear to have affected our results, as all T1-T2 correlations were large and statistically significant despite the small sample size, and visual inspections of scatterplots depicting T1 vs. T2 scores confirmed that there were no outliers driving these large correlations. We further note that the purpose of the test-retest analysis was to verify that adolescents are able to answer the SMASI items consistently over time, as opposed to generating point estimates of means or effect sizes that are meant to generalize to larger samples or other populations, and the small test-retest sample we were able to collect demonstrated that adolescents are capable of answering the SMASI reliably over time.

### Limitations

The present manuscript has several additional limitations worth noting. First, we did not present evidence that the new measure predicts health outcomes in line with the expectations of MST. Given the breadth of decisions and data that led to the production of the final SMASI measure, the equally comprehensive analyses to validate the measure are presented in an accompanying work (Goldbach et al., 2017). As described in the accompanying manuscript, both the lifetime and 30 day SMASI measures were indeed found to be significantly associated with concurrent measures of depressive symptoms, suicidality, self-harm, youth problem behaviors, and use of alcohol, tobacco, prescription drug, and illicit drug use, but were only moderately associated with a measure of general adolescent stress and had explanatory utility for those outcomes above and beyond general stress (Goldbach et al., 2017). Second, although the majority of the measure performed well across demographic subgroups and the overall measure of the 54 required items demonstrated excellent reliability in the entire sample and by subgroup, the SMASI's performance is less than ideal in some areas (e.g., subgroup internal consistency scores for negative expectancies, and homonegative communications). Some of these decrements in performance may be understandable, even predictable, based on context—for example, 17-year-old participants might be expected to respond less consistently as a group to the negative expectancies measure, because many (but not all) of them may have already had some of the experiences captured by this measure, such as coming out to friends or being rejected once their sexual identity is known by others. These past experiences likely affect their expectations about future experiences and thus their responses to this part of the measure. Furthermore, given diminishing cell sizes, it was not possible to examine the psychometric performance of the SMASI within combinations of multiple demographic subgroups (e.g., age group differences by race and ethnicity). This will be an important area to investigate in future large-scale samples that can permit more granular examinations of scale performance by multiple minority statuses.

Additionally, as with all self-report data, youth might not have accurately represented their experiences due to recall or response bias, poor comprehension, or survey fatigue. Although the survey was delivered anonymously online to all but 20 participants and great efforts were taken to limit the collection of identifiers and preserve the separation of information we collected for compensation purposes, such as email addresses, from the survey data, youth may nonetheless have been concerned about the safety of their information and declined to provide or altered their responses as a result. Although we do not have an a priori reason to believe any of the potential self-report biases materially affected the final measure, it is an important limitation of the data to acknowledge, particularly without a comparison sample or the ability to collect more objective measures of participants' experiences.

## Conclusion

In conclusion, the present study met its stated goal of developing a psychometrically sound measure of minority stress among diverse SMA. In addition to being a valuable contribution to the literature on minority stress, in which measurement of the key theoretical construct still lags behind many other areas of psychology (Morrison et al., 2016), the present study also provides a field guide to rigorous de novo measure development. By using multiple sources of information to construct our candidate item set, including qualitative studies with the population of interest and a modified Delphi process to incorporate the opinion of leading scholars in multiple relevant theoretical domains, we were able to develop a truly comprehensive set of minority stress experiences. This provided a strong foundation for the basis of measure refinement through multiple analytic approaches.

Furthermore, we introduced IRT methods, particularly measurement invariance analyses, as a lens to examining item functioning across different demographic subgroups at the item reduction stage, an innovation that can potentially preempt the development of measures that are unlikely to perform well in subsequent studies of diverse samples. The inconsistency of results from study to study and across different research labs, highlighted in the “reproducibility crisis” of recent years (e.g., Open Science Collaboration., 2015), is a noted concern to the field (Ioannidis, 2012; Baker, 2016). These inconsistencies may be attributable, in part, to measure development that was conducted based on limited or homogenous samples. Prior work has demonstrated that poor or inconsistent measurement of psychological constructs can lead directly to inconsistent results (e.g., Peterson et al., 1985; Hulleman et al., 2010). Conversely, we propose that the incorporation of multiple methods to guide item selection, as in the comprehensive framework described here, are one avenue toward addressing the problem of inconsistency across studies. By improving psychological construct measurement through the use of more rigorous methods, we can subsequently improve our understanding of psychological effects—across all areas of research, well beyond minority stress.

## Ethics statement

This study was carried out in accordance with the human subjects protection guidelines of the National Institutes of Health (NIH) and the principles of the Declaration of Helsinki. All participants in this observational survey study were minors between the ages of 14–17, and therefore provided assent to participate by verbally assenting to study procedures after reading a paper assent form document (if recruited in person) or by indicating their assent with a binary survey item after reading the online assent form outlining study procedures (if recruited online). Given that participants were sexual minorities (e.g., lesbian, gay, or bisexual) who may not have previously disclosed their orientation to their parents, and thus notifying parents about their study participation could have placed participants at additional risk, a waiver of parental consent was granted by the approving IRBs. We also obtained a Certificate of Confidentiality from the NIH. The study protocol was approved by the Institutional Review Boards of Children's Hospital Los Angeles and the University Park campus of the University of Southern California.

## Author contributions

SS and JG jointly conceptualized the study reported in this manuscript. SS was responsible for data management, developed the analytic plan, drafted the initial manuscript, and critically revised the manuscript. JG was responsible for participant recruitment and data collection, contributed to the introduction and preliminary studies sections, and critically revised the manuscript. MRM conducted the data analysis, contributed to the statistical analysis and results sections, and critically reviewed the manuscript. All authors approved the final manuscript as submitted.

### Conflict of interest statement

The authors declare that the research was conducted in the absence of any commercial or financial relationships that could be construed as a potential conflict of interest.
