# Supplementary material for: Development of the Sexual Minority Adolescent Stress Inventory
Source: Front Psychol. 2018 Mar 15;9:319. doi: 10.3389/fpsyg.2018.00319 (PMC5862853; doi:10.3389/fpsyg.2018.00319)
Supplement: Supplementary file 1 [file DataSheet1.DOCX]

Supplementary Table 1.

*14-factor EFA*

|  | Eigenvalue | Variance % |
| --- | --- | --- |
| 1. Work | 44.987 | 44.105 |
| 2. Social Marginalization | 6.560 | 6.431 |
| 3. Family Rejection | 3.757 | 3.683 |
| 4. Internalized Homonegativity | 3.068 | 3.008 |
| 5. Identity Management | 1.930 | 1.892 |
| 6. Homonegative Climate | 1.672 | 1.639 |
| 7. Intersectionality | 1.590 | 1.558 |
| 8. Negative Disclosure Experiences | 1.482 | 1.453 |
| 9. Religion | 1.418 | 1.391 |
| 10. [Eliminated – one item] | 1.252 | 1.228 |
| 11. Homonegative Communication | 1.157 | 1.134 |
| 12. [Eliminated – beliefs vs. experiences] | 1.107 | 1.085 |
| 13. Negative Expectancies | 1.045 | 1.025 |
| 14. [Eliminated – concealment] | 1.033 | 1.013 |
